# Elevated Monocyte to Lymphocyte Ratio and Increased Mortality among Patients with Chronic Kidney Disease Hospitalized for COVID-19

**DOI:** 10.3390/jpm11030224

**Published:** 2021-03-22

**Authors:** Ramsés Dávila-Collado, Oscar Jarquín-Durán, Andrés Solís-Vallejo, Mai Anh Nguyen, J. Luis Espinoza

**Affiliations:** 1 Department of Emergency Medicine, Baptist Hospital of Nicaragua, Managua 11001, Nicaragua; ramsesalidavilacollado@gmail.com (R.D.-C.); oscarjarquinduran@gmail.com (O.J.-D.); andresolis1996@gmail.com (A.S.-V.); 2 Department of Hematology and Respirology, Kanazawa University, Kanazawa 920-0942, Japan; ngmaianh2008@gmail.com; 3 Faculty of Health Sciences, Kanazawa University, Kanazawa 920-0942, Japan

**Keywords:** COVID-19, chronic kidney disease, monocytes-to-lymphocyte ratio, infection complications

## Abstract

Chronic kidney disease (CKD) constitutes a major health problem and one of the leading causes of death worldwide. Patients with CKD have impaired immune functions that predispose them to an increased risk of infections, as well as virus-associated cancers and a diminished vaccine response. In this study, we aimed to identify clinical and laboratory parameters associated with in-hospital mortality in patients evaluated in the department of emergency (ER) and admitted with the diagnosis of severe acute respiratory syndrome (SARS) caused by coronavirus disease 2019 (COVID-19) at the Baptist Hospital of Nicaragua (BHN). There were 37 patients with CKD, mean age 58.3 ± 14.1 years, admitted to BHN due to COVID-19, and among them, 24 (65.7%) were males (*p* = 0.016). During hospitalization, 23 patients with CKD (62.1%) died of complications associated with COVID-19 disease, which was a higher proportion (odds ratio (OR) 5.6, confidence interval (CI) 2.1–15.7, *p* = 0.001) compared to a group of 70 patients (64.8% males, mean age 57.5 ± 13.7 years) without CKD admitted during the same period in whom 28.5% died of COVID-19. In the entire cohort, the majority of patients presented with bilateral pneumonia, and the most common symptoms at admission were dyspnea, cough, and fever. Serum levels of D-dimer, ferritin and procalcitonin were significantly higher in patients with CKD compared with those without CKD. Multivariate analysis revealed that CKD, age (>60 years), and hypoxia measured in the ER were factors associated with increased in-hospital mortality. Among patients with CKD but not in those without CKD (OR 36.8, CI 1.5–88.3, *p* = 0.026), an increased monocytes-to-lymphocyte ratio (MLR) was associated with higher mortality and remained statistically significant after adjusting for confounders. The MLR measured in the ER may be useful for predicting in-hospital mortality in patients with CKD and COVID-19 and could contribute to early risk stratification in this group.

## 1. Introduction

Severe acute respiratory syndrome coronavirus 2 (SARS-CoV-2) which causes the novel coronavirus disease 2019 (COVID-19) has spreading through the world to become a global pandemic, causing millions of documented infections and more than two million deaths (https://www.worldometers.info/coronavirus/ (accessed on 28 January 2021)). It has become evident that although the pathogen is capable of infecting individuals in all age groups, of all ethnicities, there is high inter-individual variability in terms of disease severity and risk of death among the infected individuals [1,2]. From the beginning of the pandemic, higher death rates and more hospitalizations have been observed among men than women across all age groups [3]. In addition, epidemiological and clinical evidence indicates that older adults and people of any age who have underlying medical conditions, such as hypertension, diabetes, obesity immunocompromised state, liver disease chronic obstructive pulmonary disease (COPD), and cerebrovascular disease, have increased risk to develop severe COVID-19 disease and increased mortality rates [4].

Chronic kidney disease (CKD), defined as the presence of kidney damage or an estimated glomerular filtration rate (GFR) less than 60 mL/min per 1.73 square meters, persisting for three months or more irrespective of cause, is a disorder associated with important morbidity and mortality and is listed among the leading causes of death worldwide [5]. In 2017, more than 690 million cases of all-stage CKD were recorded worldwide, with a global prevalence of 9.1% (8.5% to 9.8%) resulting in more than 35 million deaths [6]. The primary cause of CKD varies by region and setting, with hypertension and diabetes being the most common causes [7]. In some areas of the world, especially in developing countries, in a substantial proportion of patients with CKD, the underlying cause remains unknown, and proposed etiological factors include exposure to toxins or heavy metals, as well as pathogen infections [5,7].

Increased risk of infectious complications and more adverse outcomes have been documented in patients with CKD compared with the general population [8,9], which has been attributed to the presence of severe immune dysfunctions associated with this disease. This includes the emergence of premature immunological aging with loss of thymic function, attrition of telomeres, and epigenetic changes in hematopoietic stem cells, which ultimately results in impaired immune responses [10,11]. Along with their elevated risk of severe infections, patients with CKD often have endothelial dysfunction, enhanced coagulation, and commonly have chronic comorbidities such as diabetes, hypertension, and coronary artery disease, which makes the CKD population especially vulnerable to severe COVID-19 [9,10,11,12]. Indeed, during the current COVID-19 pandemic, several challenges associated with the clinical care of CKD patients have been identified, including, problems with disease monitoring, disruption of the management and application of kidney replacement therapies, delayed or cancellation of kidney transplants, as well as more adverse clinical outcomes [13,14,15].

The clinical features of COVID-19 have been well characterized and various laboratory parameters, including the white blood cell count (WBC), C-reactive protein concentration (CRP), interleukin-1, interleukin-6, d-dimer, among others are utilized in the clinical practice for stratifying COVID-19 patients into risk groups [1,2]. In addition, prognostic scores for COVID-19 severity or mortality have been reported, but most of those scores require the use of multiple high-cost tests that may not be available in all clinical settings [16,17]. On the other hand, various studies have reported alterations in circulating blood cells in COVID-19 patients and some of these changes appear to be associated with disease prognosis. For example, lymphopenia was associated with disease severity and worse prognosis in a meta-analysis [18], and neutrophils to lymphocyte ratio (NLR) was found to have a good predictive value for disease severity in patients with COVID-19 infection [19]. Another study also found significant differences in WBC between community-acquired pneumonia and COVID-19 pneumonia, and lymphopenia and low eosinophil counts were associated with worse outcomes [20]. In this study, we aimed to identify clinical or laboratory parameters detectable in the department of emergency (ER) that may be associated with in-hospital mortality in patients admitted with the diagnosis of COVID-19, including patients with CKD. The study emphasized those clinical and laboratory indicators that are broadly available in ERs, even in healthcare units with limited resources, such as those in developing countries which are typically hampered by chronic lack of resources.

## 2. Materials and Methods

This retrospective cohort study included adult patients who were consecutively evaluated in the department of emergency of the Baptist Hospital of Nicaragua (BHN) and admitted with the diagnosis of COVID-19 between April to August 2020. BHN is a general hospital located in Managua, the capital city of Nicaragua, that serves the nationwide population, and contains a range of medical specialty departments, including a kidney care clinic with a national reference hemodialysis unit for the support and treatment of patients with CKD. Data from a total of 107 patients, including 37 patients with CKD and 70 patients without CKD, were available for this analysis. Before the current COVID-19 pandemic, the majority of the patients with CKD included in this study were being followed up, on an outpatient basis, at the kidney care unit of BHN, with the majority of them receiving three hemodialysis sessions per week. During the COVID-19 outbreak, these CKD patients were attended in the ER of BHN, which accounts for the relatively high proportion of CKD patients included in this cohort.

### 2.1. Measurements and Definition

Hospital admission was defined as the presence of a patient in the hospital for more than 24 h after being triaged in the ER and diagnosed as COVID-19 illness based on symptoms and radiology and confirmed by laboratory studies using nasopharyngeal swabs followed by polymerase chain reaction (PCR) for SARS-CoV-2. CKD definition and stage was done according to the criteria of the Kidney Disease Outcomes Quality Initiative (KDOQI) defined as the presence of kidney damage or an estimated GFR less than 60 mL/min per 1.73 square meters, persisting for three months or more irrespective of cause [21]. End-stage kidney disease (ESKD) was defined as a decrease in kidney function, expressed by a GFR of less than <15 mL/min per 1.73 m^2^ [22].

### 2.2. Data Collection and Variables

Epidemiological, clinical and laboratory data from patients with COVID-19, including those with CKD who were admitted to the BHN were sourced and extracted from the hospital information system. Only patients that were admitted with the diagnosis of COVID-19 through the ER of BHN within the study period were included in the study. Institutional Review Board approval was not required for this observational, retrospective study of routinely transmitted patient information, and written informed consent was waived owing to the rapid emergence of this infectious disease.

### 2.3. Laboratory Data and Imaging Studies

Venous blood samples of all patients were obtained in the ER and blood parameters were measured by the clinical laboratory of BHN. Blood cell count and differential were measured with an ABX Pentra XL 80 automated cell counter system (Horiba Ltd., Kyoto, Japan). The following parameters were further calculated based on leukocyte counts. Neutrophils to lymphocytes ratio (NLR), was calculated by dividing the absolute neutrophil count (ANC) by the absolute lymphocyte count (ALC); monocyte to lymphocyte ratio (MLR) was determined by dividing the absolute monocyte count (AMC) by the ALC, and the platelet to lymphocyte ratio (PLR) was calculated by dividing the absolute platelet count (APC) by ALC, as described before [23,24]. Additional blood examinations included liver function tests, ferritin, magnesium, C-reactive protein, lactate dehydrogenase, D-dimer, and procalcitonin, although these parameters were not available in all patients. A chest radiograph and chest computer tomography were obtained at baseline and as determined clinically on a case-by-case basis.

### 2.4. Statistical Analysis

Categorical variables were described as the total number and percentages and continuous variables were described as either the means with standard deviations (SDs) or median interquartile range (IQR). Normally distributed continuous variables were compared using the Student’s *t*-test and non-normally distributed continuous variables were compared with the Mann–Whitney U test. Categorical variables were compared with the chi-square test. Multivariable logistic regression was performed to model the association of demographic characteristics, laboratory data, and comorbidities with in-hospital mortality. First, we made use of univariate logistic regression models, and then the variables with a significant statistical difference were included in a multivariate analysis using the Cox logistic regression method. Odds ratios (ORs) with 95% confidence intervals (CIs) were reported for all models. Data analysis was performed using Stata version 16.1 (Stata Corp, College Station, TX, USA) and statistical significance was set at a *p*-value of ≤0.05.

## 3. Results

During the study period, 107 COVID-19 patients were admitted to the BHN with the diagnosis of COVID-19 and among them, 37 (37.5%) had CKD. The mean age of the entire COVID-19 patient cohort was 57.5 years old (SD ± 13.7 years, range 21–83 years), and among them, 70 (65.4%) were male.

In the entire cohort, most patients (79.9%) had at least one comorbidity, including arterial hypertension (HTA), 68 patients (63.5%), diabetes, 49 patients (45.7%), heart failure, 6 patients (5.6%), and asthma, 3 patients (2.8%). Patients with CKD were more likely to have HTA, diabetes and, heart failure compared with patients without CKD. Among patients with CKD admitted at BHN with the diagnosis of SARS-COVID-19, 25 (67.5%) were with ESKD and were on hemodialysis but there were no differences in mortality rate among patients with ESKD and those without ESKD (*p* = 0.15). The baseline characteristics comparing patients with CKD and without CKD (Non-CKD) at hospital admission are shown in Table 1.

As shown in Table 2, the most common symptoms at presentation were fever, dyspnea, fatigue, and cough. Some patients also presented with diarrhea and vomiting, dysarthria, and dizziness with no significant differences between patients with CKD and those without CKD (Non-CKD).

**Figure 1:**
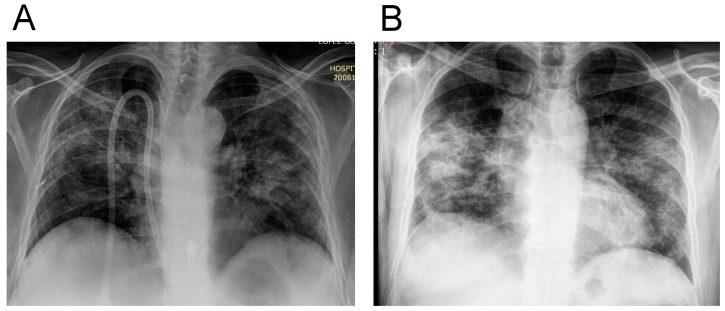
Representative chest X ray images from two patients ((**A**) CKD: chronic kidney disease and (**B**) Non-CKD: patients without CKD) admitted with the diagnosis of severe acute respiratory syndrome coronavirus disease 2019 (SARS-COVID-19) both patients presented with bilateral infiltrates and ground-glass opacifications.

The majority of patients presented bilateral pneumonia with bilateral infiltrates on chest radiographs or CT scans, with some of them presenting consolidation, ground-glass opacifications, and linear opacities (Figure 1).

Absolute leukocyte counts were higher in patients without CKD compared with those with CKD, although the differences were non-statistically significant. Patients without CKD also tended to have higher numbers of lymphocytes and platelets and, as expected, anemia was more common among patients with CKD. Notably, serum levels of d-dimer, ferritin, and procalcitonin, which have been associated with increased disease severity and higher mortality in patients with COVID-19, were significantly higher in patients with CKD, compared with those without CKD (Table 3). Unfortunately, in a considerable fraction of patients (both, in CKD and without CKD patients), these parameters were not available for this study, thus it was not possible to determine the impact of these differential parameters on patient survival by further analysis due to the limited number of data.

To identify risk factors for mortality in the studied population we performed linear logistic regression. Among the tested variables, only age, CKD, urea, hypoxia (oxygen saturation < 92% at admission), anemia (Hb < 11 g/dL), and having HTA were associated with increased risk of death. In a multivariate logistic regression model (excluding urea due to multicollinearity effect), with the exception of anemia and HTA, these factors remained statistically significant associated with increased mortality (Table 4).

**Figure 2:**
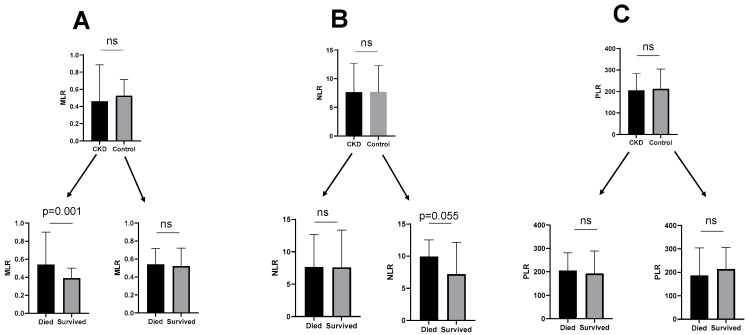
MLR (**A**), NLR (**B**), and PLR (**C**) among patients admitted with CKD (chronic kidney disease) or without CKD (control) that were admitted with the diagnosis of COVID-19. Among patients with CKD, levels of MLR were higher in the deceased patients than in those who survived ((**A**), lower panel). NLR levels were somewhat higher in patients without CKD (control) who died in hospital compared with those who survived ((**B**), lower panel). PLR values did not correlate with the survival outcome in both groups (**C**). ns: non-significant. NLR (neutrophil to lymphocyte ratio), PLR (platelets to lymphocyte ratio), MLR (monocytes to lymphocyte ratio).

High levels of blood biomarkers such as NLR, PLR, and MLR have been reported in certain inflammatory conditions, including various types of infections [23,25,26]. Notably, in comparison with previous data from a study that included 500 healthy individuals with a similar background (Hispanic population), whose normal values are as follow: PLR, mean SD 125.4 ± 36.3, median 120; NLR, mean SD 1.80 ± 0.65, median 1.70; and MLR, mean SD 0.23 ± 0.07, median 0.21 [24], the levels of these three inflammatory markers (NLR, MLR, and PLR) among the hospitalized patients included in this study were considerably higher (Table 3). Hence, a potential association between PLR, NLR, and MLR levels with in-hospital mortality among patients with CKD and those without CKD was assessed. When comparing the entire population, there were no statistically significant differences in MLR levels between patients with CKD and those without CKD (Figure 2A, upper panel), however, as shown in the lower panel of Figure 2A, among patients with CKD, significantly higher levels of MLR were observed in the deceased patients compared with those who survived (*p* = 0.001) but such differences were not observed among non-CKD patients. NLR levels were somewhat higher among patients without CKD (control) who died in hospital compared with those who survived (Figure 2B). Conversely, PLR did not correlate with the survival outcome in both groups (Figure 2C).

Next, a subgroup analysis was performed in an attempt to identify potential risk factors that could differentially affect mortality among patients with CKD or without CKD. Logistic regression showed than in patients with CKD only MLR was associated with mortality risk (OR 24.9 CI 95% 1.34–46.19, *p* = 0.03), thus substantiating the relevance of MLR as a predictor of mortality among patients with CKD hospitalized with the diagnosis of COVID-19. Conversely, in patients without CKD, age, urea, and hypoxia were found to be associated with an increased risk of mortality and both urea and hypoxia remained statistically significant after multivariate analysis (Table 5).

## 4. Discussion

This study describes the demographic and clinical characteristics of patients with CKD and those without CKD that were and admitted with the diagnosis of COVID-19 in the BHN. The in-hospital mortality rate in the entire cohort was 40% and was significantly higher among patients with CKD compared with those without CKD (62% vs. 28%), which is consistent with previous observations that patients with COVID-19 harboring CKD have worse clinical outcomes [15,27]. However, with the exception of HTA, other comorbidities such as diabetes and asthma, did not correlate with the mortality rate associated to COVID-19 in the studied population.

Infection constitutes an important cause of hospitalization among individuals with CKD, being the second leading cause of admission after cardiovascular disease [28]. In addition, mortality rates caused by infections are higher in patients with CKD compared with the general population [29], and the risk of death increases exponentially with the decrease in renal function [30]. Factors that may predispose these patients to infections include the coexistence of medical conditions such as diabetes, cardiovascular disease, advanced age, malnutrition, immunosuppression, and the presence of vascular access devices. Data from a large study showed that low serum levels of vitamin D were associated with an elevated risk of infectious events and all-cause mortality [31], and these observations have been also corroborated in other populations, including elderly adults and the general population [28]. In line with these observations, several studies have reported that vitamin D supplementation has protective effects on reducing respiratory infection in the general population [32]. These findings have crucial importance because vitamin D deficiency is a common finding among patients with CKD, especially those undergoing dialysis [33]. Interestingly, striking low levels of vitamin D have been documented in severe COVID-19 patients, and vitamin D deficiency correlated with a more pronounced inflammatory response in those patients [34]. In addition, in patients with CKD, an array of immune dysfunctions have been documented. This includes and impaired function of T and B lymphocytes and a lower phagocytic activity of neutrophils and macrophages [23,24]. Furthermore, patients with CKD requiring renal replacement therapy, repeated dialysis sessions may expose them to a possible contaminated environment. Therefore, given the above factors, patients with CKD are more vulnerable to COVID-19 than the general population and on top of that, the development of COVID-19 may worsen the impaired kidney function and further lead to rapid deterioration of kidney function and even death.

In the present study, median NLR, MLR, and PLR in the entire studied population were higher compared with reference values in healthy individuals, which is consistent with previous observations that those blood biomarkers are altered in patients with COVID-19. However, when assessing their potential impact on patients’ survival, only an elevation in MLR consistently correlated with increased mortality among patients with CKD but not in those without CKD. Since NLR, MLR, and PLR can be easily calculated from blood cell counts, these parameters have been used as potential surrogate biomarkers in several infectious and inflammatory conditions [23]. In particular, an increased monocyte to lymphocyte ratio (MLR) has been reported in association with several types of infection [25], autoimmune disorders [35], acute and chronic cardiovascular events [36], and cancer [37]. Numerous studies have reported alterations in peripheral blood cells in COVID-19 patients in association with disease severity and some of those studies evaluated the use of WBC and the absolute cell number of blood cell lineages, as well as cell-to-cell ratios including the NLR, MLR, and PLR described above, and other variations such as the lymphocyte-to-neutrophil ratio (LNR) and neutrophil-to-monocyte ratio (NMR). Recent meta-analyses have shown that lymphopenia [18] and an elevated NLR are associated with poor prognosis in patients with COVID-19 [19,38], and functional studies indicate that neutrophil [39] and monocyte activation [40] plays a central role in the pathogenesis of severe COVID-19, which substantiate the relevance of NLR and MLR as a predictor of disease severity. In a cohort of 119 Italian patients, higher values of NLR, NLPR, NLR, and the systemic immune-inflammation index (SII) were associated with in-hospital mortality due to COVID-19. However, after adjusting for confounders, only the SII, which is based on neutrophil, platelet, and lymphocyte counts, remained significantly associated with survival [41]. Another study conducted in Turkey assessed the predictive value of blood markers obtained in the ER from 233 COVID-19 patients. CRP, PLR, NLR, and lactate dehydrogenase, were significantly higher in patients with PCR-documented SARS-CoV-2 infection compared with those without SARS-CoV-2, while a higher number of eosinophil, lymphocyte, and platelet were observed among patients negative for SARS-CoV-2 [42]. Of note, in that study, the impact of these markers on in-hospital mortality was not assessed and comparisons were made between SARS-CoV-2 positive and negative patients [42]. In a cohort of 54 Mexican adult patients with COVID-19, an LNR lower than 0.088 and an NMR greater than 17.75 measured at the time of hospital admission were independent risk factors for in-hospital mortality in patients with severe COVID-19 [43]. As mentioned early, several studies have reported impaired clinical outcomes, including more intensive care unit (ICU) admissions and increased mortality among CKD patients with COVID-19. To the best of our knowledge, this is the first study showing an association between elevated MLR and higher in-hospital mortality in CKD patients admitted with COVID-19, although further studies including a large number of patients are needed to confirm these results, however, the MLR has the potential to serve as a rapidly measurable and cost-effective marker of in-hospital mortality in CKD patients with severe COVID-19.

In this study, we also noticed that dimer D, ferritin, and procalcitonin levels were significantly higher in patients with CKD compared with non-CKD patients, although these laboratory measurements were not available in a considerable fraction of patients, which impede us from carrying out further analysis to determine the impact of those serum markers in patients survival; however, it is plausible that those biomarkers do correlate with disease severity and a more severe inflammatory response. Indeed, elevated levels of d-dimer, ferritin, and procalcitonin have been associated with severe COVID-19, and poorer outcomes, including a higher mortality rate [44]. In line with these observations, previous studies have reported that patients with CKD often have higher levels of inflammatory markers such as interleukin-1b, interleukin-1RA, interleukin-6, tumor necrosis factor-alpha, and CRP, which were associated with increased risk of infection [28,45]. Notably, impaired kidney function is also associated with the emergence of markers for endothelial dysfunction compared to healthy controls and thus, endothelial dysfunction may be a contributing factor for the development of thrombosis and other cardiovascular events observed in patients with severe COVID-19 [1,2,4,46], which may account for the higher mortality in patients with CKD. Therefore, although speculative, due to the limited data, the elevation of inflammatory markers such as dimer D, ferritin, and procalcitonin, and MLR, in patients with CKD is consistent with the notion that a dysregulated immune response coupled with the presence of endothelial dysfunction contribute to the disease severity and worse outcomes in patients with CKD.

Some limitations were associated with this study. Besides the relatively small size of the population studied, the study was conducted in a single hospital in Nicaragua and the findings may not be generalizable to other populations. Importantly, the impact of other variables such as body mass index, liver disease, and immunosuppression state, which have been shown to affect disease outcomes in patients with COVID-19 [1,2,4] were not assessed in this study, as required indicators were not available in a substantial fraction of patients. In addition, this study did not take into account additional variables derived from the in-hospital treatment, such as the use of antiviral therapy, corticosteroids, ivermectin, and other drugs, as well as admission in the intensive care unit, etc. The main goal of the study was to identify factors, including clinical and laboratory biomarkers, measurable in the ER and with the potential to contribute to predicting survival in patients admitted for COVID-19.

## 5. Conclusions

This study confirms the higher risk of death among patients with CKD hospitalized with COVID-19 compared with those without CKD and also showed that the presence of an elevated MLR in CKD patients with COVID-19 was associated with increased mortality.

## Figures and Tables

**Table 1:** Main demographic characteristics of study subjects.

	CKD	Non-CKD	*p*
Gender *n* (%)			
Male	24 (65.7)	46 (64.8)	0.93
Female	13 (34.3)	24 (35.2)	
Age, years, mean (SD)	58.3 ± 14.1	57.5 ± 13.7	0.94
Number of comorbidities *n* (%)			
0	2 (5.4)	21 (30)	0.003
1	14 (37.9)	29 (41.4)	0.71
2	17 (45.9)	18 (25.7)	0.03
≥3	4 (10.8)	2 (2.9)	0.08
Types of comorbidities *n* (%)			
Diabetes	21 (67.5)	28 (40)	0.09
Arterial hypertension	33 (89.1)	35 (50)	0.00006
Heart failure	5 (13.5)	1 (1.4)	0.009
Asthma	1 (2.7)	2 (2.8)	0.9
Others	3 (8.1)	5 (7.1)	0.85
Survived *n* (%)	14 (37.8)	50 (71.4)	
Died *n* (%)	23 (62.2)	20 (28.6)	0.001
Total	37	70	

**Table 2:** Signs, symptoms and main clinical findings at admission.

Variable	CKD	Non-CKD	*p*
Dyspnea *n* (%)	23 (62.1)	53 (75.7)	0.14
Fatigue *n* (%)	17 (45.9)	39 (55.7)	0.33
Cough *n* (%)	10 (24)	28 (40)	0.18
Fever *n* (%)	14 (21.6)	18 (25.7)	0.63
Diarrhea/vomiting *n* (%)	12 (32.4)	8 (11.4)	0.008
Headache *n* (%)	5 (13.5)	10 (14.2)	0.91
Other *n* (%)	5 (13.5)	9 (12.8)	0.92
Median (IQR) hosp. time	5 (3–8)	6 (3–11)	0.52
Resp. rate Median (IQR)	22 (21–25)	24 (22–26)	0.13
Heart rate, beat/min. Median (IQR)	88 (79–96)	90 (82–103)	0.13
Temp. Median (IQR)	36.4 (36.2–36.9)	36.7 (36–37.2)	0.64
Pulse oximetry %. Median (IQR)	94 (90–96)	94 (89–97)	0.55
Bilateral pneumonia *n* (%)	35 (94.5)	65 (92.8)	0.72

IQR = interquartile range. Resp. rate: respiratory rate (breaths/min); hosp. time: time from hospital admission to outcome, days; Temp: temperature (°C), CKD (chronic kidney disease), and Non-CKD (patients without CKD).

**Table 3:** Laboratory data.

Analyte	CKD	Non-CKD	*p*	Reference Values
WBC (/µL) median IQR	7250 (6925–14,175)	10,100 (8925–16,800)	0.33	4500–10,800
Neutrophils (/µL) median IQR	8256 (5742–12,250)	8658 (6604–13,941)	0.59	
Lymphocytes (/µL) median IQR	1022 (728–1409)	1227 (967–1697)	0.35	
Platelets (µL) median IQR	237,000 (137,000–330,500)	238,000 (196,750–311,500)	0.10	150,000–450,000
Monocytes (µL) median IQR	521 (388–727)	580 (443–818)	0.29	
Hematocrit % (mean ± SD)	34.85 ± 5.9	42.75 ± 6.3	0.0001	
Hemoglobin g/dL (mean ± SD)	11.3 (9.4–12.6)	13.5 (12.5–14.8)	0.00001	
NLR (median IQR)	7.64 (5.4–12.7)	7.69 (4.5–12.3)	0.50	
PLR (median IQR)	205.6 (158–265)	212.5 (126–304)	0.97	
MLR (median IQR)	0.56 (0.29–0.88)	0.52 (0.34–0.71)	0.71	
C reactive protein mg/L (median IQR)	160 (79–284)	177 (99–278)	0.53	0–10
D-Dimer (ng/mL) median (IQR) Missing (%)	1575 (598–2510) 19 (51)	565 (264–1069) 36 (52)	0.005	Up to 500
Ferritin (ng/mL) median (IQR) Missing (%)	2065 (598–2510) 23 (62)	866 (563–1450) 43 (61)	0.04	28–365
Procalcitonin (ng/mL) median (IQR) Missing (%)	1.35 (0.53–5.71) 16 (43)	0.2 (0.1–0.33) 25 (35.7)	0.0001	0–0.5

IQR = interquartile range, NLR (neutrophil to lymphocyte ratio), PLR (platelets to lymphocyte ratio), MLR (monocytes to lymphocyte ratio). CKD (chronic kidney disease), Non-CKD (patients without CKD).

**Table 4:** Univariate logistic analysis and multivariate logistic regression.

Variable	Unadjusted OR	95% CI	*p*-Value	Adjusted OR *	95% CI	*p*-Value
Age (>60 years)	1.03	1.2–1.7	0.020	1.04	1.03–1.7	0.047
Gender	1.16	0.5–2.6	0.71	-	-	-
CKD	4.10	1.7–9.5	0.001	5.6	2.1–15.7	0.001
Diabetes	1.22	0.5–2.6	0.60	-	-	-
HTA	2.26	0.9–5.2	0.05	1.1	0.9–3.3	0.8
comorbidities	2.3	0.8–6.1	0.08	-	-	-
WBC	0.62	0.9–1.0	0.80	-	-	-
Lymph.	1.00	0.9–1.0	0.20	-	-	-
Neut.	1.01	0.9–1.0	0.70	-	-	-
Monoc.	1.52	0.5–4.9	0.48	-	-	-
Plt.	0.99	0.9–0.1	0.32	-	-	-
Hto.	0.95	0.9–1.0	0.18	-	-	-
NLR	1.06	0.9–1.0	0.58	-	-	-
PLR	0.99	0.9–1.0	0.96	-	-	-
MLR	3.01	0.7–12.1	0.10	-	-	-
CRP	1.01	0.9–0.1	0.20	-	-	-
Urea (mg/dL)	1.11	1.1–1.2	0.001	-	-	-
Hypoxia	4.24	1.8–9.6	0.001	5.2	2.0–13.5	0.001
Low Hb	3.75	1.4–9.8	0.008	3.01	0.8–10.1	0.075

* Adjusted for age, CKD, and hypoxia. Only those variables which were significant in unadjusted simple regression were considered in multiple regression analysis. HTA = arterial hypertension, CRP = C reactive protein, Lymph = Lymphocyte count, CKD = chronic kidney disease, WBC = White blood cells, Neut. = neutrophil count, Monoc. = monocyte count, Hto = Hematocrit, Plt = platelet, NLR (neutrophil to lymphocyte ratio), PLR (platelets to lymphocyte ratio), MLR (monocytes to lymphocyte ratio), LowHb (hemoglobin < 11 g/dL), OR (odd ratio), CI (confidence interval).

**Table 5:** Univariate and multivariate logistic regression (subgroup analysis).

	Non-CKD						CKD					
Variable	Unadjusted OR	95% CI	*p*-Value	Adjusted OR	95% CI	*p*-Value	Unadjusted OR	95% CI	*p*-Value	Adjusted OR	95% CI	*p*-Value
Age	1.06	1.1–1.2	0.011	1.04	1.0–1.4	0.062	0.9	0.9–1.0	0.65	0.94	0.9–1.2	0.31
Gender	1.3	0.4–4.1	0.6	-	-	-	1.04	0.2–4.1	0.95	-	-	-
Diabetes	0.7	0.2–2.2	0.56	-	-	-	0.97	0.2–3.7	0.97	-	-	-
HTA	1.5	0.5–4.5	0.3	-	-	-	1.75	0.2–14.0	0.59	-	-	-
comorbidities	0.4	0.2–2.5	0.59	-	-	-	1.69	0.09–29.4	0.71	-	-	-
NLR	1.0	0.9–1.0	0.9	-	-		1.08	0.9–1.2	0.17	-	-	-
PLR	0.9	0.9–1.0	0.5	-	-		1.0	0.9–1.0	0.13	-	-	-
MLR	1.7	0.4–12.6	0.5	-	-	-	24.9	1.34–46.7	0.031	36.8	1.5–88.3	0.026
CRP	1.0	0.9–1.0	0.13	-	-		1.0	0.9–1.0	0.27	-	-	-
Urea (mg/dL)	1.3	1.1–1.6	0.008	1.2	1.1–1.5	0.031	1.0	0.9–1.0	0.56	-	-	-
Hypoxia	7.76	2.2–26.8	0.001	6.2	1.7–23.12	0.006	1.9	0.5–7.5	0.33	-	-	-
Low Hb	2.76	0.5–15.0	0.23	-	-	-	1.3	0.9–1.75	0.21	-	-	-
creatinine	2.03	0.7–5.2	0.14	-	-	-	1.04	0.9–1.1	0.52	-	-	-

Factors associated with in-hospital mortality among patients admitted with COVID-19. Only those variables which were found to be statistically significant in univariate linear regression were considered in multiple regression analysis. HTA = arterial hypertension, CRP = C reactive protein, Lymph = Lymphocyte count, CKD = chronic kidney disease, Non-CKD= patients without CKD, WBC = White blood cells, Neut. = neutrophil count, Monoc. = monocyte count, Hto = Hematocrit, Plt = platelet, NLR (neutrophil to lymphocyte ratio), PLR (platelets to lymphocyte ratio), MLR (monocytes to lymphocyte ratio), LowHb (hemoglobin < 11 g/dL). OR (odd ratio), CI (confidence interval).

## Data Availability

The datasets generated during and/or analyzed during the current study are available from the corresponding author (J.L.E.) on reasonable request.
